# Multisystem inflammatory syndrome in adults (MIS-A): cardiovascular manifestations and in-hospital outcomes from two tertiary centers

**DOI:** 10.3389/fcvm.2026.1893335

**Published:** 2026-08-26

**Authors:** Nazima Zarubekova, Gaukhar Kurmanova, Almira Akparova, Maira Melis, Almagul Kurmanova, Amina Abdrakhmanova, Sholpan Sadykova, Ademi Samadin, Anna Shin, Zhamilya Zhankina

**Affiliations:** 1 Faculty of Medicine and Healthcare, Al-Farabi Kazakh National University, Almaty, Kazakhstan; 2 Department of Biology, Faculty of Natural Sciences, Friedrich-Alexander University, Erlangen-Nürnberg, Erlangen, Germany

**Keywords:** cardiac dysfunction, case series, coagulopathy, COVID-19, echocardiography, gastrointestinal symptoms, hyperinflammation, MIS-A

## Abstract

**Background:**

Multisystem inflammatory syndrome in adults (MIS-A) is a rare, post-infectious hyperinflammatory condition characterized by multiorgan involvement and substantial cardiovascular morbidity. This study aimed to characterize the cardiovascular phenotype and clinical outcomes of adults with MIS-A and to compare findings between ICU and non-ICU patients.

**Methods:**

We conducted a multicenter retrospective case series of 22 hospitalized patients with a MIS-A phenotype across two university-affiliated clinical bases in Almaty, Kazakhstan (City Hospital No. 1 and City Hospital No. 7). Data were retrospectively abstracted from completed inpatient medical records after discharge or in-hospital death. Demographics, clinical manifestations documented during hospital days 0−3, baseline laboratory markers (first 24 h), treatments, and outcomes were extracted from medical records.

**Results:**

The median age was 43 years (IQR, 29−63; range 19−86), and 13/22 patients were female (59.1%). Fever (≥38 °C) was documented on 18/22 (81.8%). Mucocutaneous findings included rash in 12/22 (54.5%), enanthema in 10/22 (45.5%), and non-purulent conjunctivitis in 5/22 (22.7%). Echocardiography was available for 17/22 (77.3%); LVEF <50% was observed in 6/17 (35.3%). Gastrointestinal symptoms occurred in 7/22 (31.8%), neurologic manifestations in 8/22 (36.4%), and respiratory manifestations in 16/22 (72.7%). Inflammatory markers were markedly elevated, including C-reactive protein (median 181 mg/L, IQR, 95.4–245.0) and D-dimer (median 6.4 µg/mL FEU, IQR, 4.0–10.0). Leukocytosis occurred in 15/22 (68.2%), thrombocytopenia in 11/22 (50.0%), and lymphopenia in 8/22 (36.4%). Systemic corticosteroids were used on 14/22 (63.6%); ICU admission occurred on 11/22 (50.0%). In-hospital mortality was 5/22 (22.7%).

**Conclusion:**

Hospitalized patients with a MIS-A phenotype demonstrated severe hyperinflammation with frequent multiorgan involvement and meaningful in-hospital mortality. Early recognition and standardized assessment of organ dysfunction, particularly cardiovascular involvement, are essential.

## Introduction

Multisystem inflammatory syndrome in adults (MIS-A) is a rare, delayed hyperinflammatory condition that can occur after SARS-CoV-2 infection and is characterized by fever, markedly elevated inflammatory markers, and dysfunction of multiple extrapulmonary organ systems, most commonly the cardiovascular and gastrointestinal systems. The Centers for Disease Control and Prevention (CDC) surveillance definition emphasizes hospitalization, fever within the first 3 days, laboratory evidence of inflammation, multisystem involvement, evidence of current or recent SARS-CoV-2 infection or exposure, and the absence of an alternative plausible diagnosis (1).

The epidemiology of MIS-A remains incompletely characterized, and its true incidence is difficult to determine. Reported estimates vary widely across studies, ranging from approximately 0.2% to 11.7% among populations evaluated after SARS-CoV-2 infection (2). However, these figures should be interpreted cautiously because most available studies are retrospective, use heterogeneous case definitions, and may include patients initially classified as having recurrent or biphasic COVID-19. Earlier reports predominantly described MIS-A in younger adults, particularly men, although cases across a broad age range have been documented. Geographic reporting has also been uneven, with most published cases originating from Europe and the Americas and comparatively limited clinical data available from Asian populations (2, 3).

Although the precise pathogenesis of MIS-A remains incompletely understood, current evidence suggests that it represents a post-infectious immune-mediated hyperinflammatory syndrome rather than persistent viral infection. Dysregulated activation of the innate and adaptive immune systems, excessive cytokine production, endothelial injury, and immune-mediated multiorgan inflammation are considered central mechanisms contributing to cardiovascular dysfunction and injury to multiple organ systems. These immune abnormalities typically develop several weeks after acute SARS-CoV-2 infection, supporting the concept of a delayed post-infectious inflammatory response (2–4).

The clinical relevance of MIS-A is driven by its association with critical disease and mortality in adults. Although MIS-A appears to be less common than MIS-C, adults often present with a more severe phenotype, including shock, myocardial dysfunction, thromboinflammatory complications, and overlap with severe acute COVID-19 or sepsis-like syndromes (4–10).

Comparative literature suggests that children with MIS-C more frequently demonstrate mucocutaneous findings such as rash and conjunctivitis, whereas adults with MIS-A more often present with gastrointestinal symptoms, cardiovascular dysfunction, hypotension, and need for intensive care (4, 6, 8, 9). This overlap with acute COVID-19 and other hyperinflammatory states contributes to diagnostic uncertainty in adult patients (8, 11, 12).

Early reports suggested that MIS-A often presents with shock or hypotension and myocardial dysfunction, while respiratory symptoms may be absent or mild; confirmation of antecedent SARS-CoV-2 infection frequently requires serologic testing or careful epidemiologic history because RT-PCR may be negative at presentation (3, 6, 13–15).

Cardiac involvement is particularly important because SARS-CoV-2-associated hyperinflammation may affect the myocardium through inflammatory, endothelial, thrombotic, and microvascular mechanisms. Reviews of COVID-19-related cardiac injury and myocarditis support the need for early cardiac assessment in patients with unexplained systemic inflammation after SARS-CoV-2 exposure (16–18).

Data describing MIS-A from Central Asia remain extremely limited. Furthermore, few studies have compared the clinical characteristics of ICU and non-ICU patients with MIS-A. Therefore, this study aimed to characterize the cardiovascular phenotype and clinical outcomes of adults with MIS-A treated at two tertiary care centers, with particular emphasis on differences between ICU and non-ICU patients.

## Methods

Study design and setting. We conducted a multicenter retrospective case series across two university-affiliated clinical bases in Almaty, Kazakhstan: City Hospital No. 1 and City Hospital No. 7. We included consecutive eligible admissions between 2024 and 2025. Data were retrospectively abstracted from completed inpatient medical records after discharge or in-hospital death.

Eligibility and case definition. We screened hospitalized patients with suspected post-COVID-19 hyperinflammation and included consecutive patients in whom a probable MIS-A diagnosis was established clinically and confirmed by multidisciplinary consensus after exclusion of competing diagnoses. For transparency, we assessed concordance with key elements of the CDC MIS-A surveillance definition: (1) age ≥21 years, (2) hospitalization ≥24 h (or disease/condition resulting in death), (3) fever criterion, (4) no alternative plausible diagnosis, (5) qualifying clinical criteria within days 0–3 (at least three criteria including at least one primary criterion), (6) laboratory evidence of systemic inflammation, and (7) laboratory evidence of SARS-CoV-2 infection (positive NAAT, antigen, or serology) when documented. Epidemiologic history (contact with confirmed COVID-19 and/or a recent COVID-19–compatible disease) was recorded as supportive evidence, particularly when laboratory documentation was incomplete. Cases with incomplete concordance but a convincing MIS-A clinical and laboratory phenotype (e.g., near-adult age) were retained for descriptive analysis and are reported separately (1, 6, 11, 19).

Diagnostic adjudication and exclusion of alternative diagnoses. All patients were reviewed by the supervising professor (clinical immunologist/infectious diseases specialist). The final diagnosis was established by a multidisciplinary team (consilium) including an infectious diseases specialist, clinical immunologist, neurologist, otorhinolaryngologist, surgeon, allergist, cardiologist, pulmonologist, intensive care specialist, and ophthalmologist. Diseases with a similar clinical presentation were systematically excluded. Potential autoimmune diseases were excluded through multidisciplinary clinical assessment, review of medical history, physical examination, routine laboratory investigations, and targeted immunological testing when clinically indicated. No patient was considered to have an alternative autoimmune disorder explaining the clinical presentation. To support sepsis exclusion, blood and bacterial cultures were obtained during the diagnostic workup; when available from the initial evaluation, results did not identify an alternative bacterial sepsis source. For the purposes of this study, severe cardiovascular involvement was defined by the presence of one or more of the following: myocardial dysfunction on echocardiography, cardiogenic shock requiring vasoactive support, clinically significant arrhythmias requiring treatment, or elevated cardiac biomarkers associated with acute cardiac injury.

Data sources and collection. Data were extracted from electronic medical records using a standardized form. Variables included: demographics; comorbidities; epidemiologic history (documented contact with a person with confirmed COVID-19, prior COVID-19-like disease, prior confirmed SARS-CoV-2 infection when available, and vaccination status); presenting symptoms and CDC clinical criteria assessed during days 0–3; microbiologic and serologic testing (including nasopharyngeal RT-PCR and antibody testing when available); laboratory tests within the first 24 h (and peak values when available); imaging and cardiac evaluation (electrocardiography and transthoracic echocardiography); treatments and organ support; in-hospital complications; and outcomes. All enrolled patients fulfilled the CDC diagnostic criteria for MIS-A. Patients with only suspected MIS-A who did not meet the complete case definition were not included in the study.

**Figure 1:**
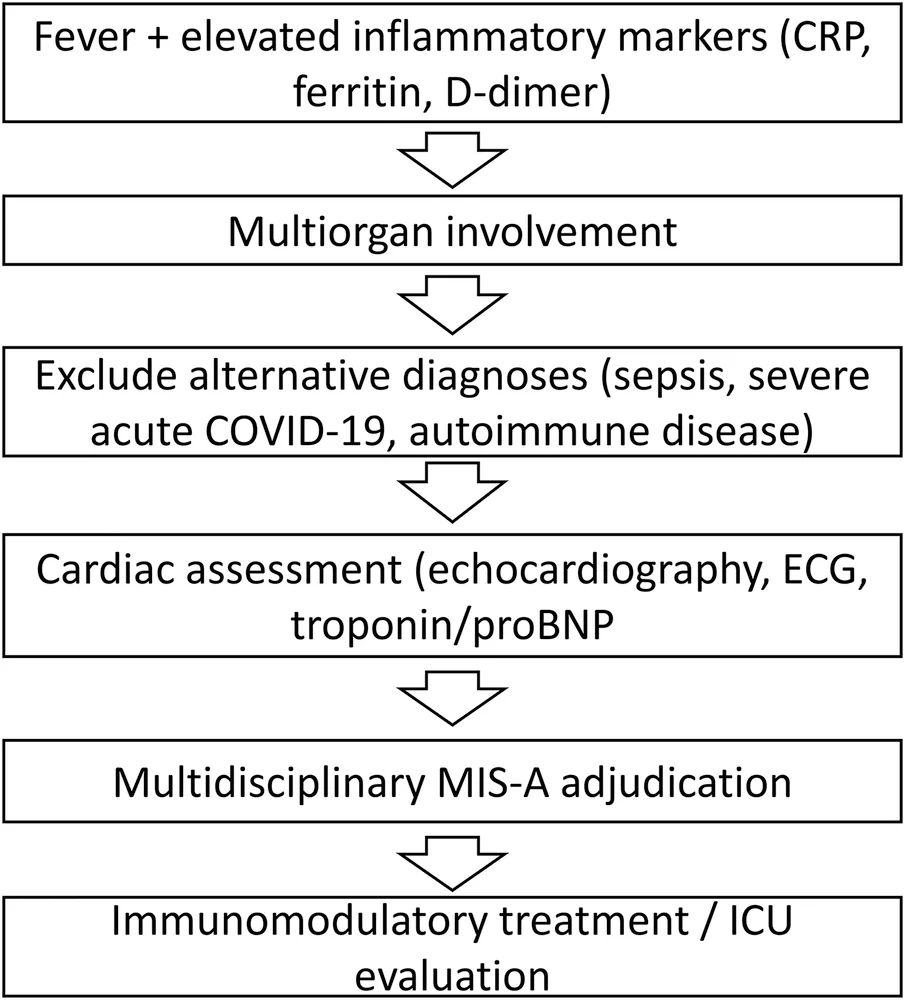
Diagnostic workflow for suspected MIS-A.

Definitions and diagnostic workflow (Figure 1). In suspected MIS-A, the intended initial mandatory workup at our centers included: clinical examination; complete blood count; inflammatory markers (CRP, ESR and/or procalcitonin when available); coagulation/injury markers (including D-dimer and fibrinogen); basic biochemical testing (renal and liver function, electrolytes, lactate as available); electrocardiography; and transthoracic echocardiography (requested as a mandatory cardiac assessment in suspected cases). Chest imaging and other targeted studies were performed according to the presenting syndrome. Epidemiologic evidence considered during adjudication included documented close contact with a confirmed COVID-19 case, serologic evidence of prior infection, and/or a recent illness clinically compatible with COVID-19. RT-PCR samples were obtained from the nasopharynx; results obtained later in the disease course (typically 5–6 days after symptom onset) were interpreted cautiously, as PCR may already be negative at the time of MIS-A presentation. After a probable diagnosis was established, patients underwent additional targeted investigations to exclude mimicking conditions and to define organ involvement.

Statistical analyses were primarily descriptive. Continuous variables were assessed for distribution characteristics and are presented as median and interquartile range (IQR), and, where informative, mean and range. Categorical variables are presented as number and percentage (*n*/*N*, %). Given that the CDC surveillance criteria specify timing before hospitalization or within the first three hospital days, primary clinical criteria were abstracted and summarized for hospital days 0–3.

Comparisons between Intensive Care Unit (ICU) and non-ICU groups were performed using the Mann–Whitney U test for continuous variables and Fisher exact test for categorical variables, as appropriate. A sensitivity review excluding the two patients aged <21 years was performed; clinical interpretation and direction of findings were unchanged.

We additionally reported the proportion of clinically diagnosed cases with complete vs. incomplete concordance for the CDC age component and summarized the availability of laboratory evidence of SARS-CoV-2 infection and other CDC surveillance elements based on routine chart documentation.

All statistical analyses and graphical visualizations were performed using GraphPad Prism version 10. A two-sided *p* value <0.05 was considered statistically significant.

Ethics. This retrospective study used routinely collected, de-identified clinical data from two university-affiliated clinical bases in Almaty, Kazakhstan (City Hospital No. 1 and City Hospital No. 7). At hospitalization, patients or their legal representatives provided general consent for inpatient care according to institutional procedures. The study protocol was reviewed and approved by the local Institutional Review Board/Ethics Committee in accordance with institutional requirements. The requirement for additional study-specific informed consent was waived because of the retrospective design and the use of de-identified data.

## Results

**Figure 2:**
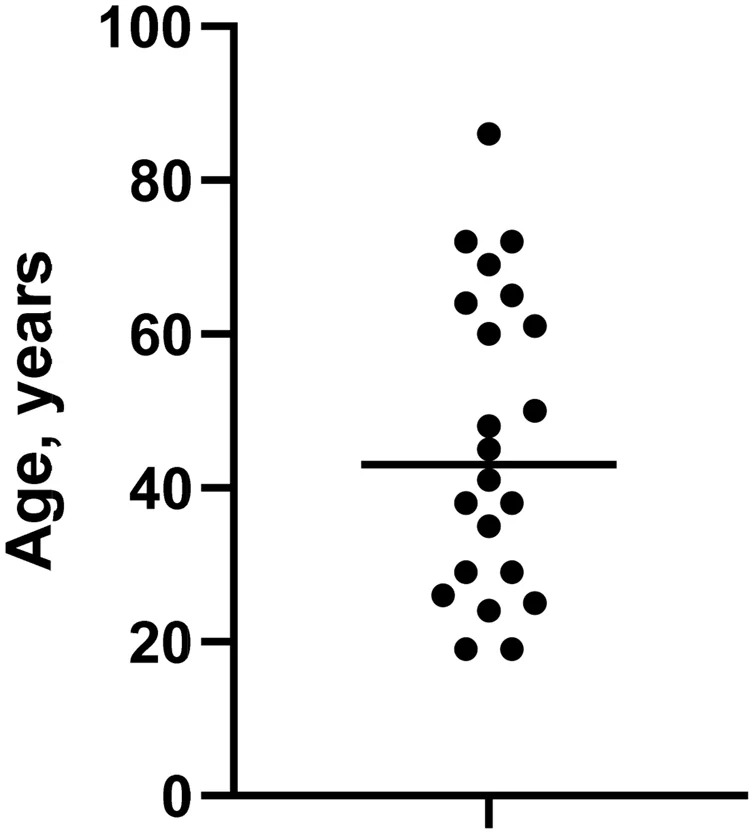
Age distribution of hospitalized patients with a MIS-A phenotype (*N* = 22).

**Figure 3:**
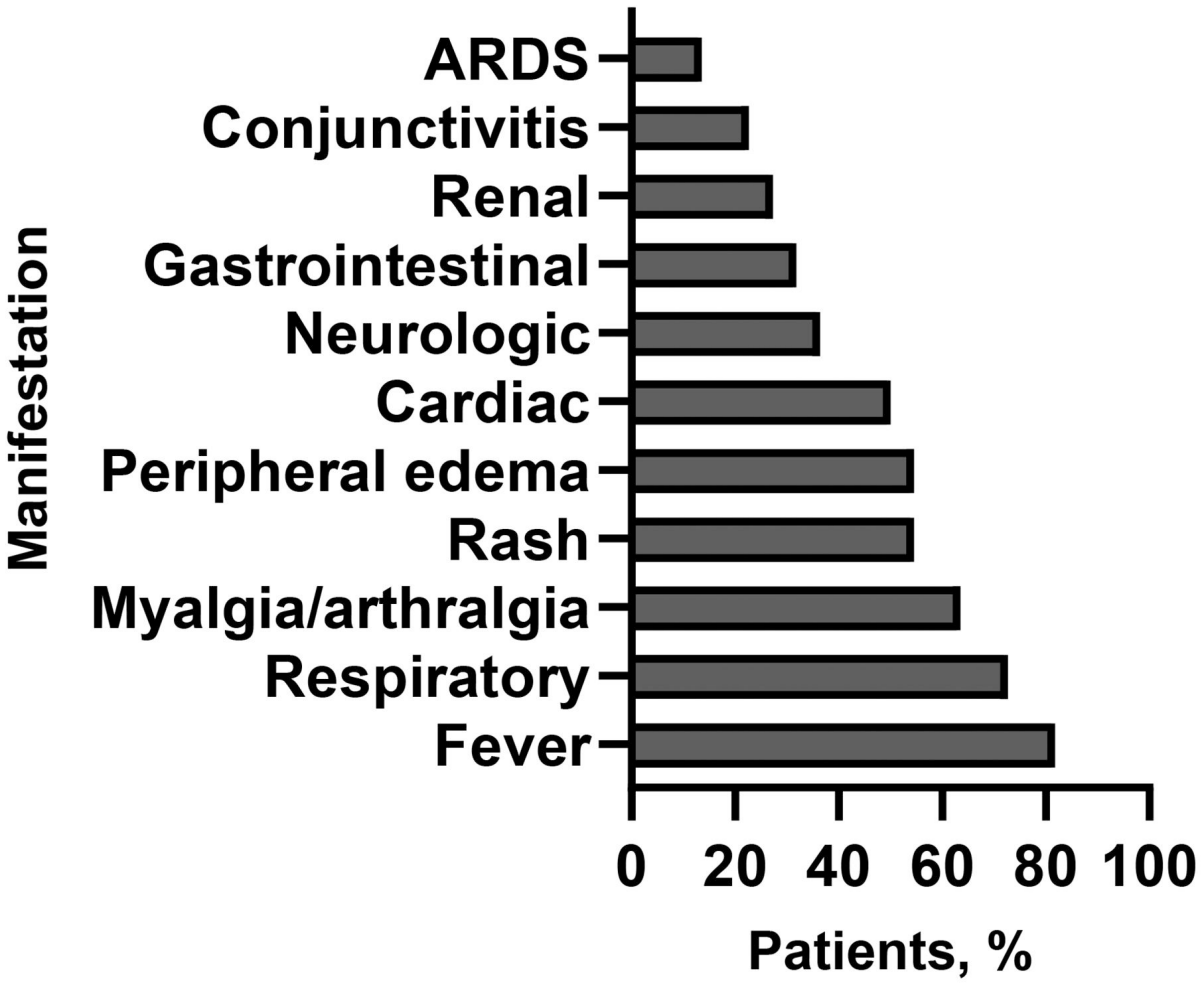
Clinical manifestations at presentation (days 0–3).

Twenty-two patients were included. The median age was 43 years (IQR, 29-63; range 19−86) (Figure 2), and 13/22 were female (59.1%). Two patients were younger than 21 years (19 years each). The median symptom duration prior to admission was 7 days (IQR, 3–14; data available for 21/22). Fever (≥38 °C) was documented in 18/22 patients (81.8%). Mucocutaneous manifestations were common: rash was recorded in 12/22 (54.5%), enanthema in 10/22 (45.5%), and non-purulent conjunctivitis in 5/22 (22.7%). Myalgia/arthralgia occurred in 14/22 (63.6%) and peripheral edema in 12/22 (54.5%). Gastrointestinal symptoms (abdominal pain and/or nausea/vomiting) were noted in 7/22 (31.8%). Neurologic manifestations were documented in 8/22 (36.4%), including peripheral neuropathy in 4/22 (18.2%) and central nervous system symptoms in 5/22 (22.7%). Respiratory manifestations (dyspnea and/or ARDS and/or abnormal chest CT) were observed in 16/22 (72.7%), and ARDS was reported in 3/22 (13.6%). Renal manifestations (hematuria and/or proteinuria and/or acute kidney injury) were documented in 6/22 (27.3%); renal/urinalysis data were available for 7 patients, of whom 6 (85.7%) demonstrated renal involvement (Figure 3).

**Figure 4:**
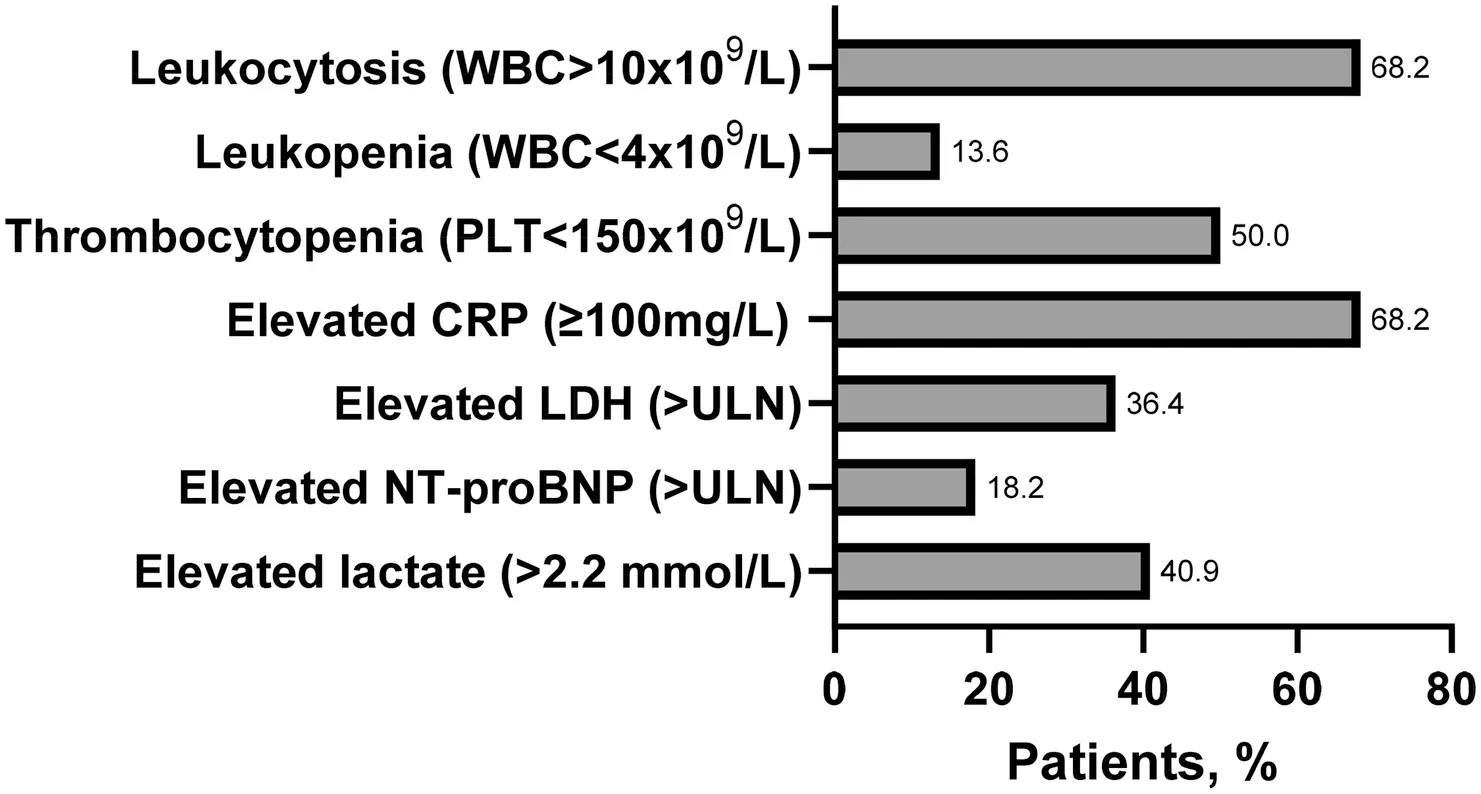
Major laboratory abnormalities at presentation (*N* = 22); numeric labels (*n*/*N*, %) are shown on bars. Numeric labels are shown on the bars; Table 1 reports exact denominators per laboratory parameter.

Baseline laboratory abnormalities are summarized in Table 1, Figure 4 and included leukocytosis in 15/22 (68.2%), leukopenia in 3/22 (13.6%), anemia in 13/22 (59.1%), thrombocytopenia in 11/22 (50.0%), and lymphopenia in 8/22 (36.4%). C-reactive protein was markedly elevated (median 181.0 mg/L, IQR, 95.4−245.0), as were D-dimer (median 6.4 µg/mL FEU, IQR, 4.0−10.0), lactate (median 2.48 mmol/L, IQR, 1.0−4.7), and procalcitonin (median 1.09 ng/mL, IQR, 0.15−8.22). In-hospital mortality was 5/22 (22.7%).

**Table 1:** Key laboratory parameters within the first 24 h of admission (available values analyzed).

Laboratory parameter	*n*	Median (IQR)
White blood cell count, ×10^9^/L	22	12.69 (5.30–19.23)
Platelets, ×10^9^/L	22	141.5 (77.0–362.5)
C-reactive protein (CRP), mg/L	21	181 (96–245)
Ferritin, µg/L	11	974 (545–2,304)
D-dimer, µg/mL FEU	17	6.4 (4.0–10.0)
Fibrinogen, g/L	20	4.76 (3.23–5.65)

Clinical severity at presentation was reclassified according to the need for ICU-level care and signs of organ dysfunction. Eleven patients (50.0%) admitted to ICU were classified as critically ill, and the remaining eleven patients (50.0%) were classified as severe. Lymphopenia, hypotension, low oxygen saturation, and organ-support requirements were considered supportive severity markers when present.

Among the 22 patients with a clinician-assigned MIS-A diagnosis, 20/22 (90.9%) met the CDC surveillance age threshold (≥21 years) (Table 2). Two patients (9.1%) were 19 years old and therefore had incomplete concordance on the age component only; both otherwise met the CDC clinical-criterion threshold and demonstrated marked systemic inflammation and were retained in the descriptive cohort and reported separately. Blood and bacterial cultures obtained during the initial evaluation to exclude bacterial sepsis, when available, did not identify an alternative bacterial sepsis source.

**Table 2:** Selected CDC clinical-criterion components identified prior to or during hospitalization (days 0–3) (*N* = 22).

Clinical criterion	*n*/*N* (%)
Severe cardiac illness/heart failure*	11/22 (50.0%)
Rash	12/22 (54.5%)
Non-purulent conjunctivitis	5/22 (22.7%)
Neurologic signs/symptoms	8/22 (36.4%)
Shock or hypotension (not medication-related)*	8/22 (36.4%)
Abdominal pain, vomiting, or diarrhea	7/22 (31.8%)
Thrombocytopenia*	11/22 (50.0%)

* Documented within hospital days 0–3.

Electrocardiography was performed in all patients and repeated during hospitalization when clinically indicated. Cardiac troponin was measured in 11 patients, of whom 7 had concentrations above the upper reference limit. NT-proBNP measurements were available for 19 of the 22 patients. Echocardiography was available for 17/22 patients (77.3%) (Table 3). Reduced left ventricular ejection fraction (LVEF <50%) was observed in 6/17 (35.3%), including severe dysfunction (LVEF <35%) in 1/17 (5.9%). Regional or global hypokinesis was documented in 7/17 (41.2%). Right ventricular systolic dysfunction was reported in 8/17 (47.1%). Pericardial effusion was noted in 1/17 (5.9%). Myocarditis was suspected on echocardiography in 3/17 (17.6%), and pulmonary hypertension in 3/17 (17.6%). Among patients with repeat LVEF assessment (*n* = 9), 7/9 (77.8%) recovered to LVEF ≥60%.

**Table 3:** Echocardiographic findings (patients with available echocardiography; *n* = 17).

Finding	*n*/*N* (%)
LVEF <50%	6/17 (35.3%)
LVEF <35%	1/17 (5.9%)
Regional/global hypokinesis	7/17 (41.2%)
Right ventricular systolic dysfunction	8/17 (47.1%)
Pericardial effusion	1/17 (5.9%)
Myocarditis suspected	3/17 (17.6%)
Pulmonary hypertension	3/17 (17.6%)
Repeat LVEF assessment available	9/17 (52.9%)
Recovered to LVEF ≥60% (among repeats)	7/9 (77.8%)

In-hospital complications were frequent and involved multiple-organ involvement (Table 4). Pulmonary complications were the most common, followed by acute-phase/sepsis-like complications and cardiac involvement. These categories were based on documented clinical diagnoses, imaging findings, laboratory abnormalities, and organ-support requirements during hospitalization.

**Table 4:** In-hospital complications (*N* = 22).

Complication category	*n*/22 (%)	Description
Cardiac complications	12/22 (54.5%)	Included myocarditis/myocardial dysfunction, pericardial involvement, rhythm abnormalities, heart failure, or cardiogenic shock.
Pulmonary complications	16/22 (72.7%)	Included pneumonia or pneumonitis, pleural effusion, respiratory failure, ARDS, or need for mechanical ventilation.
Acute-phase/sepsis-like complications	14/22 (63.6%)	Included severe systemic inflammation, shock-like presentation, suspected bacterial superinfection, or antibiotic treatment due to risk of secondary infection.
Other complications	10/22 (45.5%)	Included anemia, coagulopathy/DIC, thrombosis, renal involvement, multiorgan dysfunction, or secondary immunodeficiency.

Treatment and organ support are summarized in Table 5. Systemic corticosteroids were administered in 14/22 patients (63.6%). Vasopressors were required in 4/22 (18.2%). Invasive mechanical ventilation was used in 3/22 (13.6%), and renal replacement therapy in 3/22 (13.6%). Plasmapheresis and intravenous immunoglobulin were each used in 1/22 patients (4.5%).

**Table 5:** Treatments and organ-support measures during hospitalization (*N* = 22).

Treatment/support	*n*/*N* (%)
Systemic corticosteroids	14/22 (63.6%)
Vasopressors	4/22 (18.2%)
Invasive mechanical ventilation	3/22 (13.6%)
Renal replacement therapy (dialysis)	3/22 (13.6%)
Plasmapheresis	1/22 (4.5%)
Intravenous immunoglobulin (IVIG)	1/22 (4.5%)

Clinical outcomes are summarized in Table 6. ICU admission occurred in 11/22 patients (50.0%). Length of stay was available for 18/22 patients (81.8%), with a median of 13 days (IQR, 10−15.5; range 6−54). Prolonged hospitalization was observed in a subset of patients, including one woman who underwent cesarean delivery followed by hysterectomy on hospital day 8, contributing to an extended length of stay. Early response to treatment, as documented in the medical records, was noted in 6/22 patients (27.3%). In-hospital mortality was 5/22 (22.7%). All five non-survivors developed macrophage activation syndrome, characterized by bicytopenia with severe thrombocytopenia and anemia, hyperferritinemia, and persistent hypotension requiring prolonged inotropic support. In three patients, MAS progressed to multiorgan failure, which was the primary cause of death. In the remaining two patients, severe myocarditis with a progressive decline in left ventricular ejection fraction resulted in refractory heart failure and death.

**Table 6:** Clinical outcomes (*N* = 22).

Outcome	Value
ICU admission	11/22 (50.0%)
Length of stay, days (available *n* = 18)	median 13 (IQR, 10–15.5); range 6–54; mean 16.3
Early response to treatment (documented)	6/22 (27.3%)
In-hospital mortality	5/22 (22.7%)

Evidence of prior SARS-CoV-2 exposure or COVID-19-compatible disease was summarized in Table 7. Prior COVID-19-like symptoms were reported in 4/13 patients (30.8%) with available documentation. Most patients had a clinically resembling COVID-19 within approximately one week before admission to the university-affiliated clinical bases, according to the adjudication records. Known contact with COVID-19 was recorded in 2/12 (16.7%). Vaccination against COVID-19 was documented in 3/13 patients (23.1%); all three had received Gam-COVID-Vac (Sputnik V), a recombinant adenoviral vector vaccine. RT-PCR testing during the index hospitalization was performed in 7/22 patients (31.8%): 1/7 (14.3%) was positive and 6/7 (85.7%) were negative. Anti-spike IgG results were available for 2/22 patients (9.1%), and both tested positive.

**Table 7:** Evidence of SARS-CoV-2 exposure and COVID-19-compatible disease.

Measure	*n*/*N* (%)
Prior COVID-19 symptoms (history)	4/13 (30.8%)
Known contact with COVID-19	2/12 (16.7%)
COVID-19 vaccination documented	3/13 (23.1%)
RT-PCR performed during hospitalization	7/22 (31.8%)
RT-PCR positive (among tested)	1/7 (14.3%)
Anti-spike IgG available	2/22 (9.1%)
Anti-spike IgG positive (among tested)	2/2 (100%)

CDC criteria concordance (key surveillance elements). In the cohort with a clinician-assigned MIS-A diagnosis, 20/22 patients (90.9%) met the CDC surveillance age threshold (≥21 years). The remaining 2/22 patients (9.1%) were near-adult 19-year-old cases and therefore had incomplete concordance on the age component. Laboratory evidence of SARS-CoV-2 infection (NAAT and/or serology) was variably documented (Table 7); accordingly, we report the cohort as a clinician-adjudicated MIS-A phenotype and provide CDC element concordance and exposure/testing data transparently.

Comparison between patients requiring ICU admission and non-ICU patients is summarized in Table 8. Patients requiring ICU-level care tended to demonstrate more severe clinical presentation, including higher frequencies of thrombocytopenia, shock, and in-hospital mortality. Mortality was observed exclusively in the ICU group (45.5% vs. 0%, *p* = 0.01). No statistically significant differences were identified for age, inflammatory markers, or reduced left ventricular ejection fraction between groups, although the small sample size limited statistical power. Overall, ICU admission reflected substantial disease severity and multiorgan involvement in hospitalized patients with MIS-A phenotype.

**Table 8:** Comparison of patients requiring ICU admission vs. non-ICU patients.

Variable	ICU (*n* = 11)	Non-ICU (*n* = 11)	*P* value
Age, years	48 (19–72)	41 (24–86)	0.78
CRP, mg/L	181.0 (95.4–319.5)	170.5 (41.9–434)	0.99
D-dimer, µg/mL	5.4 (1.15–47)	6.5 (2.2–19.6)	0.94
Ferritin, µg/L	897 (206.53–3,607.0)	1,850 (194.6–9,054.0)	0.79
Thrombocytopenia, *n* (%)	7 (63.6%)	4 (36.4%)	0.2
LVEF < 50%, *n* (%)	4 (36.4%)	4 (36.4%)	1.0
Shock, *n* (%)	5 (45.5%)	3 (27.3%)	0.38
Mortality, *n* (%)	5 (45.5%)	0	0.01

Continuous variables are presented as median (IQR) and compared using Mann–Whitney U test. Categorical variables are presented as *n* (%) and compared using Fisher exact test.

## Discussion

In this multicenter case series, MIS-A presented as a severe systemic hyperinflammatory syndrome characterized by frequent cardiovascular involvement, marked inflammatory activation, and substantial morbidity. Half of the patients required intensive care, and the in-hospital mortality reached 22.7%, underscoring the potential severity of this condition. Echocardiographic abnormalities were common, including biventricular systolic dysfunction, while recovery of left ventricular ejection fraction was observed in most patients who underwent follow-up assessment, supporting the potentially reversible nature of cardiac involvement. Patients requiring intensive care exhibited a more severe clinical course, with higher rates of shock, thrombocytopenia, multiorgan dysfunction, and all recorded deaths occurring in this subgroup. Overall, our findings emphasize the heterogeneous clinical presentation of MIS-A and highlight the importance of early recognition and multidisciplinary management.

The demographic characteristics of our cohort were generally consistent with previously published reports of MIS-A (3, 4). The median age of 43 years falls within the range reported in systematic reviews, which indicate that MIS-A predominantly affects young and middle-aged adults. However, our cohort also included two 19-year-old patients and one 86-year-old patient, illustrating that the clinical phenotype may extend beyond the age groups typically emphasized in surveillance definitions. Two patients (9.1%) showed incomplete concordance on the age criterion solely because they were 19 years old. Nevertheless, both exhibited a clinical and laboratory phenotype fully compatible with the case definition, as confirmed by multidisciplinary consensus. Because the CDC definition was developed for surveillance rather than bedside diagnosis, several case-finding studies have adjusted age thresholds for adult services to capture near-adult presentations; accordingly, we retained these cases for descriptive reporting and provided concordance transparently (1, 11, 19). Although early reports suggested a male predominance (3, 6), women represented the majority of our cohort (59.1%). Similar variability in sex distribution has been observed across published case series and likely reflects the limited sample sizes, geographic differences, and heterogeneous patient populations included in available studies. These findings support the concept that MIS-A should be considered across a broad adult age spectrum regardless of sex when compatible clinical features are present.

The spectrum of clinical manifestations observed in our cohort was broadly consistent with published systematic reviews (3, 4, 6, 11, 20), which identify fever, mucocutaneous findings, gastrointestinal symptoms, and cardiovascular involvement as the predominant features of MIS-A. Although the frequency of individual manifestations varied across studies, this variability has been attributed to differences in study design, diagnostic criteria, disease severity, and referral patterns. Our findings further support the concept that MIS-A represents a multisystem inflammatory syndrome with heterogeneous clinical presentation, requiring a high index of suspicion in patients presenting with unexplained fever and multiorgan dysfunction following SARS-CoV-2 infection.

Cardiovascular involvement is considered one of the defining features of MIS-A and represents the principal cause of critical illness in affected adults. Previous systematic reviews have consistently identified myocardial dysfunction, shock, arrhythmias, and elevated cardiac biomarkers as the most prominent manifestations distinguishing MIS-A from uncomplicated COVID-19 and many other post-infectious inflammatory syndromes (3, 4, 6). In the largest systematic review by Patel et al. (6), cardiovascular involvement was reported in 87% of patients, including cardiac dysfunction in 54%, myocarditis in 30%, and pericardial effusion in 25%. Similarly, Kunal et al. (4) found cardiovascular involvement in 81% of patients, while reduced left ventricular ejection fraction was observed in 73.2% of those who underwent echocardiography. Michailides et al. (3) reported cardiac manifestations in 97.1% of patients. Likewise, Varghese et al. (20) observed cardiovascular involvement in 81% of patients, with myocarditis, reduced left and/or right ventricular ejection fraction, and hemodynamic instability representing the most common cardiovascular manifestations.

In our cohort, clinically significant cardiovascular involvement was identified in 54.5% of patients. Although clinically significant echocardiographic abnormalities were identified in approximately half of the cohort, evidence of cardiac involvement according to the CDC case definition was present in all patients, emphasizing that cardiac injury in MIS-A extends beyond structural abnormalities detected by echocardiography. Echocardiography demonstrated both left and right ventricular systolic dysfunction, supporting the concept that cardiac injury in MIS-A is frequently diffuse rather than limited to isolated left ventricular impairment. Although the prevalence of ventricular dysfunction in our cohort was lower than that reported in previous systematic reviews, direct comparisons should be interpreted cautiously because echocardiography was not performed in all patients in our study and the extent of cardiac evaluation varied considerably across published cohorts.

An important observation in our cohort was the recovery of left ventricular systolic function in most patients who underwent follow-up echocardiography. This finding is in agreement with previous reports suggesting that myocardial dysfunction in MIS-A is frequently reversible following resolution of systemic inflammation and appropriate immunomodulatory and supportive therapy (4). Such reversibility distinguishes MIS-A from many forms of chronic cardiomyopathy and supports the hypothesis that myocardial depression is predominantly inflammation-mediated rather than the consequence of irreversible structural myocardial injury.

The mechanisms of cardiac injury in MIS-A are likely multifactorial and may include cytokine-mediated myocardial depression, endothelial dysfunction, microvascular injury, and immune-mediated myocardial inflammation rather than direct viral cytotoxicity. These mechanisms are consistent with the delayed post-infectious nature of MIS-A and may explain the frequently reversible cardiac dysfunction observed in many patients (4).

These findings support routine early cardiovascular assessment, including echocardiography and cardiac biomarker evaluation, in patients with suspected MIS-A, as timely recognition of cardiovascular involvement may facilitate appropriate intensive care and immunomodulatory treatment.

The high proportion of patients requiring intensive care and the observed in-hospital mortality underscore the severity of MIS-A once multiorgan dysfunction develops. Previous systematic reviews have consistently reported that a substantial proportion of adults with MIS-A require intensive care because of circulatory shock, myocardial dysfunction, respiratory failure, or other manifestations of multiorgan involvement (3, 4, 6, 15). In the systematic review by Michailidis et al. (3), 54.9% of patients required intensive care, with an overall mortality of 5.6%. Similarly, Patel et al. (6) reported ICU admission in 57% of patients and an in-hospital mortality of 7%, while Kunal et al. (4) found that 58.1% of patients required ICU care, with a mortality rate of 5.1%.

In our cohort, 50% of patients required ICU admission, which is comparable to previous reports. However, the in-hospital mortality was substantially higher (22.7%) than that reported in these systematic reviews. This difference should be interpreted cautiously given the small sample size and differences in patient populations, referral patterns, and the extent of cardiovascular assessment across published studies.

Notably, all deaths occurred among patients admitted to the ICU, emphasizing that critical illness in MIS-A is primarily associated with progressive cardiovascular instability and multiorgan dysfunction rather than isolated inflammatory abnormalities. Patients requiring intensive care more frequently presented with shock, thrombocytopenia, and organ failure, whereas inflammatory biomarkers alone did not significantly distinguish ICU from non-ICU patients. Although the limited sample size precludes definitive conclusions, these findings suggest that early clinical evidence of organ dysfunction may be more informative for risk stratification than isolated inflammatory markers.

The absence of statistically significant differences in inflammatory biomarkers between ICU and non-ICU patients should also be interpreted with caution because of the limited statistical power. Nevertheless, this observation suggests that disease severity in MIS-A may depend not only on the magnitude of systemic inflammation but also on the extent of cardiovascular and multiorgan involvement and the host immune response.

Taken together, our findings indicate that the severe cardiovascular phenotype of MIS-A is closely associated with the need for intensive care and adverse in-hospital outcomes. These observations further reinforce the role of early cardiovascular assessment as part of the initial evaluation of patients with suspected MIS-A.

The diagnosis of MIS-A remains challenging because no single clinical feature or laboratory marker is sufficiently specific. Patients frequently present with fever, shock, myocardial dysfunction, elevated inflammatory markers, and multiorgan involvement, a clinical picture that substantially overlaps with bacterial sepsis, toxic shock syndrome, acute myocarditis, hemophagocytic syndromes, and systemic autoimmune diseases. Consequently, delayed recognition may postpone appropriate immunomodulatory treatment.

Our findings further illustrate these diagnostic difficulties. Several patients initially underwent extensive evaluation for bacterial sepsis or other infectious conditions before MIS-A was considered. Negative microbiological investigations in the absence of an alternative diagnosis, together with evidence of systemic inflammation and multisystem involvement, ultimately supported the diagnosis of MIS-A.

Based on our experience and current CDC criteria, we developed a practical diagnostic algorithm intended to facilitate the early recognition of MIS-A in hospitalized adults. Rather than replacing established diagnostic criteria, the algorithm provides a structured approach to identifying patients with compatible clinical features, excluding alternative infectious etiologies, assessing cardiovascular involvement, and integrating epidemiological evidence of recent SARS-CoV-2 infection.

The proposed algorithm may be particularly useful in centers where MIS-A is infrequently encountered and where the syndrome may not be initially suspected. A structured diagnostic approach may shorten the time to diagnosis, facilitate timely cardiological evaluation, and support earlier initiation of immunomodulatory therapy in appropriate patients.

Because the algorithm was derived from a retrospective case series, its clinical performance has not yet been prospectively validated. Future multicenter studies are needed to evaluate its diagnostic accuracy and clinical utility in broader patient populations.

Overall, our findings emphasize that MIS-A should be considered in adults presenting with unexplained hyperinflammation, shock, or acute cardiac dysfunction following recent SARS-CoV-2 infection, particularly when microbiological investigations fail to identify an alternative infectious cause. A structured diagnostic algorithm may facilitate earlier recognition of this uncommon but potentially life-threatening syndrome and improve multidisciplinary clinical decision-making.

## Limitations

Several limitations should be acknowledged. The retrospective design introduces the potential for incomplete data collection and selection bias. The relatively small sample size reflects the rarity of MIS-A and limits statistical power, particularly for subgroup analyses. Because this was an observational study without a control group, causal relationships and independent predictors of adverse outcomes could not be established. In addition, not all patients underwent uniform follow-up investigations, including repeat echocardiography or cardiac magnetic resonance imaging, which may have underestimated the full spectrum and evolution of cardiac involvement. Vaccination history was incompletely documented in routine medical records, with vaccine-related information available for only a subset of patients. Consequently, the potential influence of vaccine type on the clinical presentation or outcomes of MIS-A could not be evaluated. Finally, the proposed diagnostic algorithm has not been prospectively validated and should be considered hypothesis-generating until evaluated in larger multicenter cohorts.

## Conclusion

MIS-A is a rare but potentially life-threatening post-infectious complication of SARS-CoV-2 characterized by marked systemic inflammation, frequent cardiovascular involvement, and substantial risk of critical illness. In this multicenter case series, cardiac dysfunction, circulatory shock, and multiorgan involvement were common, with half of the patients requiring intensive care and all in-hospital deaths occurring in the ICU. Despite the severity of presentation, recovery of left ventricular systolic function in most patients with follow-up echocardiography suggests that cardiac injury is frequently reversible when recognized and treated appropriately. Our findings emphasize the importance of maintaining a high index of suspicion for MIS-A in adults presenting with unexplained hyperinflammation and multisystem dysfunction after recent SARS-CoV-2 infection. The proposed diagnostic algorithm may support earlier recognition and multidisciplinary evaluation of suspected cases, although prospective validation is required. Larger multicenter studies are needed to refine risk stratification, validate diagnostic approaches, and optimize management strategies for this uncommon but severe syndrome.

## Data Availability

The raw data supporting the conclusions of this article will be made available by the authors, without undue reservation.
